# Bioinspired Membrane-Coated Nanoplatform for Targeted Tumor Immunotherapy

**DOI:** 10.3389/fonc.2021.819817

**Published:** 2022-01-10

**Authors:** Dan Mu, Pan He, Yesi Shi, Lai Jiang, Gang Liu

**Affiliations:** State Key Laboratory of Molecular Vaccinology and Molecular Diagnostics, Center for Molecular Imaging and Translational Medicine, School of Public Health, Xiamen University, Xiamen, China

**Keywords:** bioinspired membrane, nanoparticle, tumor targeting, immunotherapy, nanobiotechnology applications

## Abstract

Immunotherapy can effectively activate the immune system and reshape the tumor immune microenvironment, which has been an alternative method in cancer therapy besides surgery, radiotherapy, and chemotherapy. However, the current clinical outcomes are not satisfied due to the lack of targeting of the treatment with some unexpected damages to the human body. Recently, cell membrane-based bioinspired nanoparticles for tumor immunotherapy have attracted much attention because of their superior immune regulating, drug delivery, excellent tumor targeting, and biocompatibility. Together, the article reviews the recent progress of cell membrane-based bioinspired nanoparticles for immunotherapy in cancer treatment. We also evaluate the prospect of bioinspired nanoparticles in immunotherapy for cancer. This strategy may open up new research directions for cancer therapy.

## Introduction

Cancer has been one of the most refractory diseases worldwide, causing millions of deaths with a vast social consumption annually (1, 2). Much progress has been made in cancer treatments, such as surgical excision, chemotherapy, and radiotherapy, and the survival of cancer patients has also been greatly improved (3–5). However, the initial clinical response rate to many tumors did not achieve the desired results, and with the extension of treatment time, the tumor often develops drug resistance and is easy to relapse. Cancer immunotherapy as a new therapy developed rapidly in recent years, which can control and kill tumor cells by stimulating or rebuilding the immune system (6). Nonetheless, it was reported that these treatments could not achieve the ideal therapeutic results and even caused unexpected damages to the human body due to deficient tumor targeting (7, 8). Therefore, there is an urgent need to develop a novel delivery system to address the issues.

**Scheme 1:**
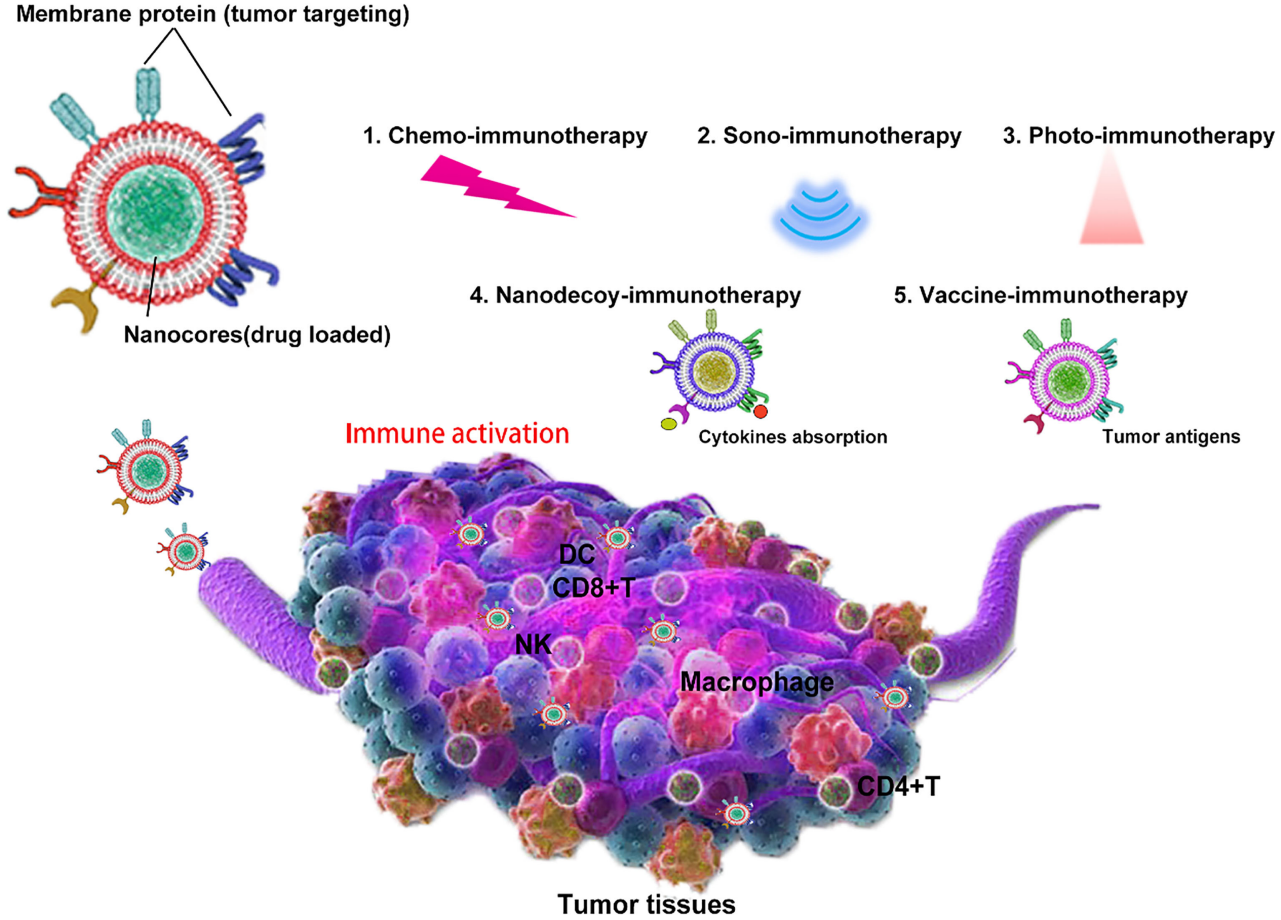
Bioinspired cell membrane-based nanoparticles in tumor immunotherapy. Cell membrane-coated nanoparticles can be used as nanodecoy-, vaccine-, photo-, sono-, and chemo-immunotherapy for tumor eradication.

Recently, cell membrane-coated nanoparticles have attracted much attention due to their biocompatibility, prolonged half-time, and superior tumor targeting from the source cells. Tumor immunotherapy based on bioinspired nanoparticles is a new therapy developed rapidly in recent years. Despite its potential significance for cancer treatment with excellent immune effect, there is a lack of discussion that focuses on bioinspired nanoparticles. Hence, this study aims to review bioinspired nanoparticles with different functions and strategies, such as nanodecoy-, vaccine-, photo-, sono-, and chemo-immunotherapy (**Scheme 1**), and also discusses the current lack of development and future development prospects.

## Cell Membrane Coating Nanoplatform in the Therapy of Cancer

According to the function of membrane-coated nanoparticles, it was briefly classified into nanodecoy-, vaccine-, photo-, sono-, and chemo-immunotherapy, and the advancement of membrane-coated nanoplatforms (NPs) in tumor treatment is also discussed in this section.

### Nanodecoy-Immunotherapy in Cancer Treatment

Tumor cells can produce various cytokines (mainly including GM-CSF, granulocyte-macrophage colony-stimulating factor, and CXCL2, chemokine ligand 2), resulting in an immunosuppressive environment through recruiting myeloid-derived suppressor cells (MDSCs) and thus inhibit the functions of tumor-specific CD8^+^ T cells and cause tumor cells’ immune escape (9, 10). Due to this fact, it remains the major obstacle that limits the efficacy of immunotherapy, such as immune checkpoint therapy. MDSCs (consisting of ∼80% PMN-MDSCs, polymorphonuclear and ∼20% M-MDSCs, monocytic populations) are responsible for the immunosuppressive tumor microenvironment (TME), which not only primarily suppresses CD8^+^ T cells’ immune response but also directly facilitates tumor growth and metastasis. Target elimination of MDSCs may help improve antitumor immune response, but it often brings about serious side effects. Recently, the pseudoneutrophil cytokine sponges (pCSs), fabricated by coating neutrophil membrane onto PLGA cores to mimic PMN-MDSCs, were reported (10). Inheriting the properties of source cells, pCSs can specifically absorb or neutralize MDSC-related cytokines and hence decrease or disrupt the recruitment of MDSCs and subsequently relieve immune tolerance. When incubated with GM-CSF and CXCL2, pCSs can show a superior binding capacity to them in a dose-dependent manner even compared with RBC@NPs. Inspired by the facts, mice bearing B16F10 were injected pCSs daily for 8 days, then the peripheral lymphoid organs and tumors were collected and analyzed by flow cytometry, and the results showed that pCSs could significantly suppress the expansion of MDSCs in the bone marrow and thus decreased their assembly in peripheral lymphoid organs and tumors. However, in immunodeficient B6/Rag1−/− or NOG mice, pCSs treatments did not limit the progress of melanoma or breast cancer. At the same time, it could delay the growth of the tumors in normal mice. In short, the antitumor activity of pCSs is established on an intact immune system. In murine breast cancer 4T1 and melanoma B16F10 models, pCSs administration can significantly enhance the infiltration of CD^+^8 T cells and improve antitumor immune response. Furthermore, in the combination therapy with anti-PD-1, pCSs suppress tumor growth and prolong survival. Collectively, the neutrophil cell membrane-coated NPs can be a novel immune-modulating nanoplatform for effective cancer immunotherapy.

### Vaccine-Immunotherapy in Cancer Treatment

Cancer vaccines can drill immune cells to specifically recognize and eradicate cancerous cells while sparing normal cells, which is established by effective tumor-associated antigen delivery (11, 12). However, application of cancer vaccines is rarely reported in clinics. Presently, tumor vaccine development is notoriously limited because tumor antigens are derived from normal antigens with subtle mutation or facile upregulation that is difficult to stimulate cellular immunity (13, 14). In particular, cancer cell membrane-coated nanoparticles have been used in homologous targeting drug delivery because of the entire inheritance of source cells. Therefore, taking advantage of cancer membrane, whose membrane proteins could also be tumor-specific antigens, to activate immune response would be a promising strategy to enhance immunotherapy (15, 16).

In a recent study, B16-F10 cancer cell membrane-coated murine-specific CpG-NPs (CpG-CCNPs) achieved a superior prophylactic and therapeutic efficacy in melanoma therapy (17). In the design, adjuvant CpG-loaded PLGA NPs were synthesized to be the inner cores, which can stimulate the maturation of DCs and the subsequent activation of tumor-specific T cells through TLR-9 signaling. When incubated with bone marrow-derived dendritic cells (BMDCs), the inner cores wrapped with the B16-F10 membrane showed more enhanced endocytosis by BMDCs compared with bare CpG NPs. Consistent with the findings, after subcutaneous injection, CpG-CCNPs can be actively internalized by macrophages and BMDCs in the draining lymph node while B or T cells had relatively less cell uptake due to the nonspecific interactions. DCs can be significantly activated to mature with the confirmation of the upregulation of CD40, CD80, CD86, and MHC-II. Meanwhile, due to the existence of melanoma major antigens such as gp100 and tyrosinase-related protein (TRP)-2 on the surface of CpG-CCNPs, it can strongly generate gp100-specific and TRP-2-specific T cells in the spleen, verifying the previous speculation that the nanoparticles were able to train the immune system against various tumor antigens. When vaccinated with CpG-CCNPs, mice then received B16-F10 cancer cell injection challenges and showed an enhanced tumor-preventing efficacy (86% of mice were tumor free during the 5-month post-challenge) compared with other formulations. Then, the therapeutic efficacy of the CpG-CCNPs was also examined in B16-F10 tumor-bearing mice, and the results revealed that subcutaneous injection of the CpG-CCNPs combined with an intraperitoneal injection of anti-CTLA-4 and anti-PD-1 could inhibit tumor growth and prolong the survival time than other treatments. Besides CpG, the toll-like receptor 7 agonist imiquimod (R837) as a novel adjuvant was also encapsulated into PLGA NPs and then covered with mannose-modified tumor cell membranes (NP-R@M-M). Significantly, the B16-OVA cancer cell membrane was wrapped onto the NPs and then intradermally injected into mice bearing B16-OVA melanoma tumor, and it can effectively trigger the maturation of DCs and subsequent specific T-cell response. Correspondingly, NP-R@M-M (B16-OVA cancer cell membrane coating) alone or combined with anti-PD-1 checkpoint therapy exhibited an enhanced B16-OVA tumor-inhibiting efficacy while sparing 4T1 breast cancer tumor, illustrating the specificity of the tumor nanovaccine. Collectively, the works provided a rational design by applying autologous cancer cell membrane as tumor-specific antigen and combining coating nanotechnology to construct an antitumor nanovaccine platform.

### Sono-Immunotherapy in Cancer Treatment

Sonodynamic therapy (SDT) is based on ultrasound (US), and it can produce large amounts of cytotoxic singlet oxygen (^1^O2) and induce US cavitation and hyperthermia (18). Due to its superiorly deeper tissue penetration, SDT has been developed as a potential alternative to traditional cancer therapy (19). Considering the fact that current SDT agents often show a low SDT efficacy due to insufficient tumor accumulation, a bioinspired membrane-coated nanoplatform would overcome these limitations (20). Moreover, SDT can also be used to activate the antitumor immune response and demonstrate a superior synergistic effect with immunotherapy.

In a recent study, a macrophage membrane-coated nanoplatform, integrating SDT, chemotherapy, and immunotherapy, is fabricated (18). In the design, production of ^1^O_2_ *in situ* and targeted delivery carbon monoxide (CO) to TME were combined upon stimulation by the exogenous US and endogenous H_2_O_2_. Other than physically inducing cancer cell death, the macrophage-coated nanoplatform can also take advantage of these cracked tumor cells to activate tumor-specific CD8^+^T cells to enhance immunotherapy. Importantly, due to the existence of the macrophage membrane, the nanoplatform can inhibit immune clearance, prolong drug circulation time, and thus enhance tumor suppression. Then, the chemotherapeutic NLG919, an indoleamine 2,3-dioxygenase (IDO) inhibitor, was loaded into the nanoparticles to inhibit tumor metastasis. Collectively, the macrophage membrane-coated nanoplatform represents a promising antitumor strategy by integrating multimode cancer therapies, which would be an alternative to clinics in the future.

### Photo-Immunotherapy in Cancer Treatment

Photodynamic therapy (PDT) is a promising cancer therapeutic strategy and has attracted much attention due to its non-invasiveness (21, 22). Local tumors can be inhibited by the reactive oxygen species (ROS) generated by PDT due to photosensitizers and laser irradiation (23), whereas the unwanted photosensitizer leakage from delivery vehicles has largely limited the progress of PDT, and PDT alone is not enough to active systemic immune response to eradicate the metastatic tumor cells (24–26). Therefore, decreasing photosensitizer leakage and improving tumor targeting would reverse the unsatisfactory therapeutic outcomes.

**Figure 1:**
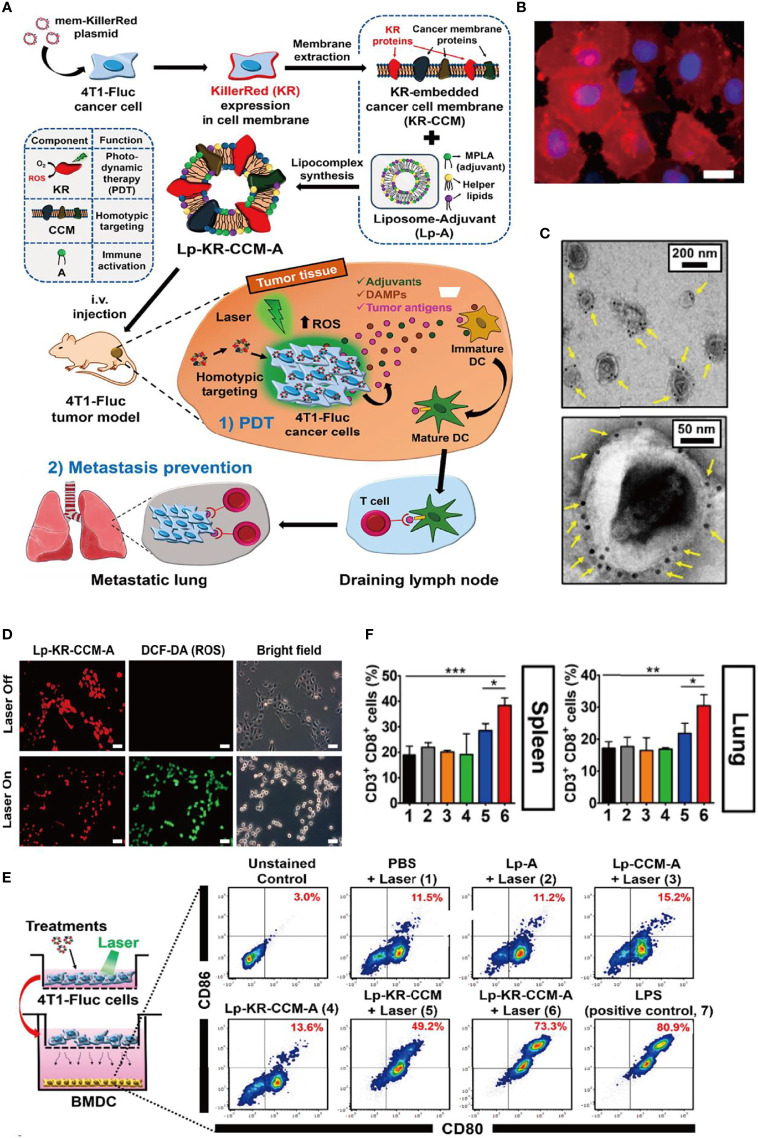
Bioinspired membrane-based nanotherapeutics for photo-immunotherapy. **(A)** Schematic illustration of preparation of Lp-KR-CCM-A and its application in cancer therapy. KR as photosensitizer in Lp-KR-CCM-A and can produce ROS upon laser irradiation to kill cancer cells (PDT) and thus enhance immunotherapy with the help of lipid adjuvant MPLA. **(B)** Representative FL image showing KR expression in 4T1-Fluc cell membrane after transfection with mem-KR plasmid. **(C)** Lp-KR-CCM-A stained with KR antibody-conjugated immuno-gold. **(D)** *In vitro* ROS generation induced by Lp-KR-CCM-A internalized in 4T1-Fluc cells upon laser irradiation for 20 min. DCF-DA was used as an indicator of intracellular ROS. **(E)** *In vitro* BMDC maturation following different treatments and irradiation of 4T1-Fluc cells in a co-culture system. **(F)** Analysis of cytotoxic CD8^+^T cells (gated on CD3^+^ T cells) in the spleen and lung after the indicated treatments. *p < 0.05; **p < 0.01; ***p < 0.001. Reproduced with permission (27), Copyright 2019, American Chemical Society.

In a recent study, Kim et al. developed a cell membrane nanovesicle-based PDT strategy and efficiently inhibit local tumor growth and suppress its metastasis (27) (**Figure 1**). Notably, KillerRed (KR), a red fluorescence protein with emission spectrum 510–600 nm and a photo-responsive sensitizer with solid ability to generate ROS upon laser irradiation, was selectively expressed on 4T1-Fluc cancer cells, avoiding the leakage mentioned above from vehicles that reversely enhanced PDT therapy. Then, the KR overexpressing cancer membrane was extracted (KR-CCM) and then hybridized with monophosphoryl lipid A (MPLA)-embedded liposomes to form the about 250-nm lipocomplex (Lp-KR-CCM-A). Especially in the design, the 4T1 cancer membrane can improve tumor targeting (about 3.3-fold higher cancer-targeting efficiency than a control liposome) because of homotypic affinity and MPLA can stimulate an immune response by targeting TLR4. After PDT, the subsequently generated ROS induced cancer cells apoptosis and the released cancer damage-associated molecular patterns (DAMPs) elicited DC maturation to active systemic tumor-specific T cells to attack the metastatic cancer cells in homotypic tumor-bearing mice. In short, the study novelty constructed the biomimetic lipocomplex technology and may improve cell membrane-based cancer therapy.

### Chemo-Immunotherapy in Cancer Treatment

Recurrence is one of the significant challenges that cause patient death even after radical surgery in cancer therapy (28). In addition, it has been reported that surgery wound and the resulting inflammatory environment may accelerate recurrence or metastasis. Hence, performing a post-operation consolidation treatment is necessary, and immune checkpoint blockade (ICB) to revert exhausted CD8^+^T cells has raised much attention (29–31). Despite significant progress, current ICB-based therapies are still restricted by autoimmune disorders and low objective drug response (32). Unwanted binding of PD-1 or PD-L1 antibody to normal tissues with i.v. injection may be one of the main reasons responsible for the compromised efficacy (33). Recently, platelet-based systems have attracted much attention as bioinspired drug delivery vehicles (28, 34). However, it potentially limited its progress and clinical use since blood-separated platelets are anucleated cellular fragments without proliferation potency (35). To address the issues, a strategy of genetic engineering platelet-based cascade amplification immunotherapy was proposed (36). In the design, lentivirus encoding EGFP-PD-1 was used to infect megakaryocyte (MK) progenitor cell line L8057 to express PD-1 stably. Stimulated by PMA, MKs underwent maturation, morphology change, and ultimately produced PD-1-expressing platelets. Due to the intrinsic properties, the purified platelets can actively target the tumor surgery wound or the resulting inflammatory microenvironment, and are then activated to produce microparticles. In the incomplete-surgery B16F10 tumor model, after three times i.v. injection of PD-1 platelets, the growth of residual tumor was significantly suppressed, whereas free platelets treatment could not prevent the recurrence. Through flow cytometry analysis, it was observed that PD-1-expressing platelets could induce more CD8^+^ T cells to infiltrate the tumors than that of free platelets or PBS, and the infiltrated CD8^+^ T cells showed enhanced secretion of granzyme B, indicating a reversion of T-cell exhaustion. The superior therapeutic outcomes can be attributed to *in situ* activation of platelet-derived microparticles of PD-1. To verify whether the *in situ* activation resulted in tumor eradication, bare aPDL1-platelet derived microparticles (PMPs) were collected from the platelets in similar research. Moreover, the results illustrated that direct injection of the PMPs could not inhibit the tumor and no more than free antibody. These results can illustrate that *in situ* activations of P–aPD-1/L1 at the tumor surgery wound were crucial for anticancer effect. Moreover, to further evaluate the ability of depletion of Tregs in TME, a model drug cyclophosphamide (CP) was loaded into PD-1-expressing platelets through co-culture or electroporation. In the therapy of the same B16F10 tumor model with incomplete resection, the results showed that CP-PD-1 platelet treatment could decrease Tregs in TME while vastly increasing the frequency of reinvigorated CD8^+^T cells, demonstrating directly blocking tumor relapse. Collectively, the study identified that gene engineering PD-1 vesicle could be an effective bioinspired multifunctional platform for cancer theranostics, in which targeted therapeutic delivery and immunotherapy were combined.

## Discussion and Future Perspective

Cancer immunotherapy changes the treatment pattern of tumors and brings hope for tumor patients, especially those with advanced malignant tumors. However, it also faces many problems, such as low immune response rate, lack of adequate and reliable predictive markers of curative effect, and lack of targeting. Monoclonal antibody immunotherapy, CAR-T, or TCR-T therapy cannot show an excellent therapeutic effect on all individuals and all tumors, and the adverse reactions are not the same. The selection of specific targets and the combined application of multiple therapies can partially solve the problem of mistarget faced by cancer immunotherapy at present (37). With the continuous emergence and innovation of photodynamic, sonodynamic, and other new technologies and methods, immunotherapy based on cell membrane-coated nanoparticles has ushered in rapid development, showing great potential for cancer treatment in the early stage of clinical trials (38, 39). However, the efficacy of cancer treatment still needs to be further improved, and future research needs to find more specific immune targets, such as tumor-specific antigens and new immune checkpoints, to avoid unnecessary targeting and missed toxicity.

In addition, future studies still need to consider the following two aspects: (1) Based on the different types and mechanisms of cell membrane-coated nanoparticle immunotherapy, how can the unique toxicity caused by histocompatibility problems in immunotherapy be avoided? (2) The discovery of cancer drugs depends on preclinical models to determine the priority of drug targets, to study the mechanism of action, the method of administration, the dose and time of treatment, and safety management (40). At present, immunotherapy based on cell membrane-coated nanoparticles is mostly limited to the essential animal experimental stage, and the clinical conversion rate is low, so the construction of a preclinical model close to the human immune environment is the key.

## Data Availability Statement

The raw data supporting the conclusions of this article will be made available by the authors, without undue reservation.

## Author Contributions

DM, PH, YS, and LJ conceptualized and wrote the manuscript. GL corresponded to the article. All authors contributed to the article and approved the submitted version.

## Conflict of Interest

The authors declare that the research was conducted in the absence of any commercial or financial relationships that could be construed as a potential conflict of interest.

## Publisher’s Note

All claims expressed in this article are solely those of the authors and do not necessarily represent those of their affiliated organizations, or those of the publisher, the editors and the reviewers. Any product that may be evaluated in this article, or claim that may be made by its manufacturer, is not guaranteed or endorsed by the publisher.
